# Delay in coronary artery bypass grafting for STEMI patients improves hospital morbidity and mortality

**DOI:** 10.1186/s13019-020-01134-x

**Published:** 2020-05-12

**Authors:** Anthony Lemaire, Tudor Vagaonescu, Hirohisa Ikegami, Lindsay Volk, Nina Verghis, Leonard Y. Lee

**Affiliations:** Division of Cardiothoracic Surgery, Department of Surgery, RUTGERS-Robert Wood Johnson Medical School, 125 Paterson Street, New Brunswick, New Jersey 08903 USA

**Keywords:** Coronary disease, Infarction, Revascularization

## Abstract

**Objectives:**

The optimal timing of coronary artery bypass grafting (CABG) in patients with ST elevated acute myocardial infarction (STEMI) is unclear. The purpose of the study is to evaluate and compare the outcomes in STEMI patients who underwent CABG within the various time intervals within the first 7 days of either emergent or urgent hospital admission.

**Methods:**

Patients aged 30 years old and older diagnosed with STEMI who underwent CABG within first 7 days after non-elective hospital admission were selected from the National Inpatient Sample 2010–2014 using the appropriate ICD-9-CM diagnosis and procedure codes. These patients were divided into 3 cohorts based on timing of surgery: within 24 h (group A), 2nd-3rd day (group B), and 4th–7th day (group C). The rates of postoperative complications, mortality, and postoperative hospital length of stay (LOS) were compared using the Chi-square test, multivariable logistic regression analysis, and Wilcoxon rank sum test.

**Results:**

A total of 5963 patients were identified: group A = 28.5%, group B = 36.1%, group C = 35.4%. Mean age overall was 63.1 ± 11.1 years; 76.9% were males and 72.9% were whites. Compared to groups B and C, patients in group A were more likely to develop cardiac complications (OR [odds ratio] =1.33, 95%CI [confidence interval] 1.12–1.59 and OR = 1.39, 95%CI 1.17–1.67, respectively) and respiratory complications (OR = 1.31, 95%CI 1.13–1.51 and OR = 1.53, 95%CI 1.32–1.78, respectively). They were also more likely to have renal complications (OR = 1.31, 95%CI 1.11–1.54) and bleeding (OR = 1.20, 95%CI 1.05–1.37) than patients in group B and had a similar tendency compared to group C. We did not find significant differences in the above complications between groups B and C. Postoperative stroke and sternal wound infection rates were similar between all three groups. In-hospital mortality was also higher in group A (8.2%) compared to group B (3.5%) and group C (2.9%, *P* < 0.0001 for both); differences between groups B and C were not significant. This was confirmed in the multivariable logistic regression analysis with controlling for age, gender, race, the Elixhauser Comorbidity Index, and complications (group A vs B: OR = 1.85, 95%CI 1.52–2.25; group A vs C: OR = 2.21; 95%CI 1.82–2.68). Patients in group A had a significantly longer postoperative LOS (median 7 days with IQR [interquartile range] 5–10 days) compared to those in group B (median 6 days, IQR 5–8 days) and group C (median 6 days, IQR 4–8 days; *P* < 0.0001 for both).

**Conclusions:**

The results of this study show that despite the urgency and severity of STEMI, patients who undergo CABG within the first 24 h after non-elective hospital admission have increased hospital morbidity and mortality. These findings suggest that a delay in surgery beyond the first 24 h may be beneficial to patient outcomes. Furthermore, there is a significant cost effectiveness when the patients delay surgery because the hospital length of stay is reduced as well as the subsequent hospital costs.

## Introduction

Acute myocardial infarction (AMI) is a major cause of death in middle aged and elderly populations. It is defined as the sudden blockage of one or more coronary arteries leading to myocardial cell death, coronary atherosclerosis, and thrombus. In the Western world, AMI is associated with significant morbidity and mortality, not only in the short term but also for many years afterwards [1]. Primary percutaneous coronary intervention (PCI) is the standard interventional treatment modality for managing patients with ST-segment elevation myocardial infarction (STEMI) [2]. There are occasions when interventional cardiologists are not able to open the culprit vessel and then surgical intervention becomes imperative. The use of PCI however, continues to be the favored option for STEMI patients. Multivessel coronary artery disease (MVD) on the diagnostic coronary angiogram, which is seen in 50–80% of patients [3, 4], presents a significant challenge for interventional cardiologists in these situations. While most agree upon addressing the culprit vessel, there is considerable debate on the timing of intervention on the other vessels.

Coronary artery bypass grafting (CABG) is normally deferred for later revascularization of the other non-culprit arteries. When the culprit vessels cannot be opened by PCI there is often pressure on cardiothoracic surgeons to operate on patients sooner who have had a STEMI. The rationale is that surgical revascularization is the only remaining option if the percutaneous approach has failed and that it needs to be performed immediately. Another perceived benefit of surgery is that full revascularization can be obtained earlier and minimize cardiac ischemia. The current views on when CABG should be performed on STEMI patients is not entirely clear. The purpose of our study is to determine the optimal timing for CABG in patients who present with STEMI.

## Materials and methods

The data for this project was obtained from the AHRQ (Agency for Healthcare Research and Quality) HCUP (Healthcare Cost and Utilization Project) Nationwide/National Inpatient Sample (NIS) for the years 2010–2014. This is the largest all-payer database in the United States covering about 20% of all hospitalizations in the acute care community hospitals that is up to 8 million for each year. The detailed information about the elements of the NIS database is available at https://www.hcup-us.ahrq.gov/db/nation/nis/nisdbdocumentation.jsp.

The study population included patients aged 30 years old and older who were hospitalized non-electively with a diagnosis of ST elevation myocardial infarction (STEMI) and underwent CABG during 7 days after hospitalization. To select these patients we used the ICD-9-CM (International Classification of Diseases, Ninth Revision, Clinical Modification) diagnosis codes 410.× 1 for the principal diagnosis to limit the sample to initial presentations of STEMI and procedure codes 36.11–36.16 for principal procedure. Using the NIS data elements PRDAY1, we categorized the whole study population into three time-groups based on the time of the CABG performance: group A (within the first 24 h), group B (2nd-3rd day), and group C (4th–7th day after admission). Patients younger than 30 years of age accounted for only 0.08% of selected cases and were excluded from analysis.

The main outcomes of our interest were postoperative complications, in-hospital mortality, post-operative and total hospital LOS (length of stay), and total hospital cost. The latter was calculated from the total hospital charges provided in the NIS, using the HCUP Cost-to-Charge Ratio Files with adjustment for inflation to 2014 dollars. The main postoperative complications were identified with the following ICD-9-CM diagnosis codes applied to the secondary diagnoses: 997.1 and 427.5 – for cardiac complications; 512.1, 512.8, 518.4, 518.5, 518.81–518.82, and 997.39 – for respiratory complications; 997.5, 584.x, 586, and 593.81 – for renal complications; 997.02, 430, 431, and 432.x – for postoperative stroke and cerebral hemorrhage; 038.xx, 785.52, 995.91, 995.92, 998.0, and 998.59 – for sepsis and bloodstream infection; 998.11, 998.12, and 285.1 – for bleeding; 998.59 – for sternal wood infection; and 415.1x – for pulmonary embolism. The use of blood transfusion was determined with the ICD-9-CM diagnosis code V58.2 and procedure codes 99.00–99.04.

SAS software (SAS Institute, Cary, NC), version 9.4 was used for data analysis and statistics. Categorical variables and difference between them were estimated with the Chi square test and the following multivariable logistic regression analysis with control for patient age, gender, race, comorbidities, and CABG time-group. Comorbidities were evaluated with the AHRQ comorbidity measures that are the NIS data elements. To control for comorbidities in the multivariable analysis of hospital mortality we used the Elixhauser comorbidity index score for in-hospital mortality that was calculated using the SAS program available on the HCUP website [5]. Difference between continuous variables was evaluated by t-test or non-parametric Wilcoxon rank sum test as appropriate after the Kolmogorov-Smirnov test for normality of distribution. A two-sided *P* < 0.05 was considered significant.

The study was approved by the Institutional Review Board of the Rutgers Robert Wood Johnson Medical School. ^1^Elixhauser Comorbidity Software, Version 3.7. Available at https://www.hcup-us.ahrq.gov/toolssoftware/comorbidity/comorbidity.jsp. Accessed January 8, 2019.

## Results

The study population included 5963 patients who met the inclusion criteria. Demographic characteristics of these patients in all three studied groups are presented in Table 1. The proportion of patients in group A (28.5%) was significantly smaller than in groups B (36.1%) and C (35.4%; *P* < 0.002 for both). The mean age overall was 63.1 ± 11.1 years; males predominated over females in all groups. Age and gender structures of patients in studied groups were similar. However, proportion of white patients in group A was lower than in group B (*P* = 0.0066) and C (*P* = 0.0008). There were no intergroup differences in proportions of blacks and Hispanics.

**Table 1:**
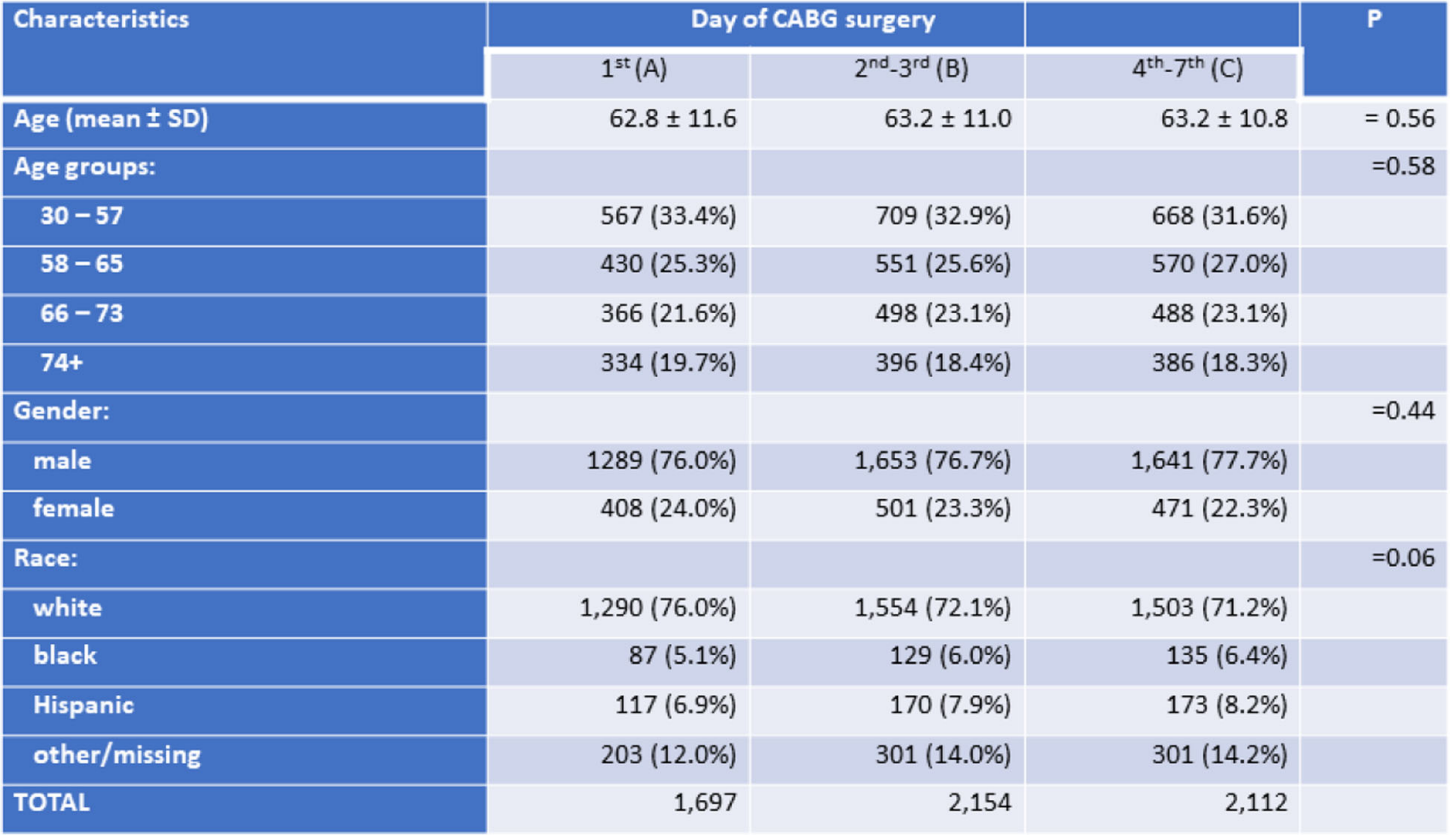
Demographic characteristics of the study population

The rates of major postoperative complications in all studied groups were measured and compared. Patients in group A had a significantly higher rate of complications overall (72.7%) than those in group B (67.4%; *P* = 0.0004) and group C (67.8%; *P* = 0.02). There was no statistically significant difference between groups B and C. In the multivariable logistic regression analysis patients in group A were still more likely to develop any postoperative complication that their counterparts in group B (OR [odds ratio] = 1.22; 95%CI [confidence interval] 1.05–1.41) and group C (OR = 1.18; 95%CI 1.01–1.36). Multivariable analysis confirmed similarity in complications overall between groups B and C (OR = 0.97; 95%CI 0.85–1.11).

**Fig. 1:**
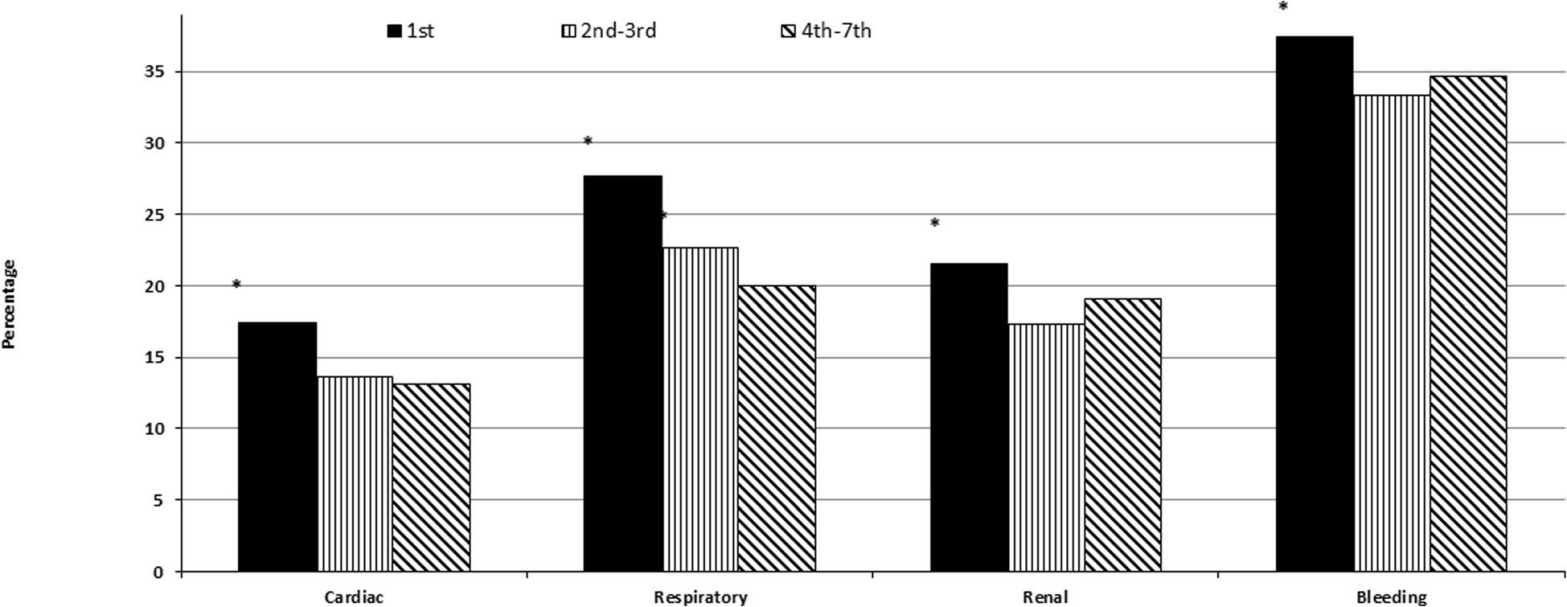
Postoperative complications

Figure 1 demonstrates specific complications with the significant variations in rates between studied groups. We found that compared to patients who underwent CABG more than 24 h after STEMI (groups B and C), patients with CABG within the first 24 h after STEMI (group A) were more likely to develop cardiac complications (OR = 1.33, 95%CI 1.12–1.59 and OR = 1.39, 95%CI 1.17–1.67, respectively) and respiratory complications (OR = 1.31, 95%CI 1.13–1.51 and OR = 1.53, 95%CI 1.32–1.78, respectively). Patients in group A were also more likely to have renal complications (OR = 1.31, 95%CI 1.11–1.54) and bleeding (OR = 1.20, 95%CI 1.05–1.37) than patients in group B and had a similar tendency compared to group C. At the same time, we did not find significant differences in the above complications between groups B and C as well as differences in the rates of postoperative stroke, pulmonary embolism, and sternal wound infection between all three groups.

**Fig. 2:**
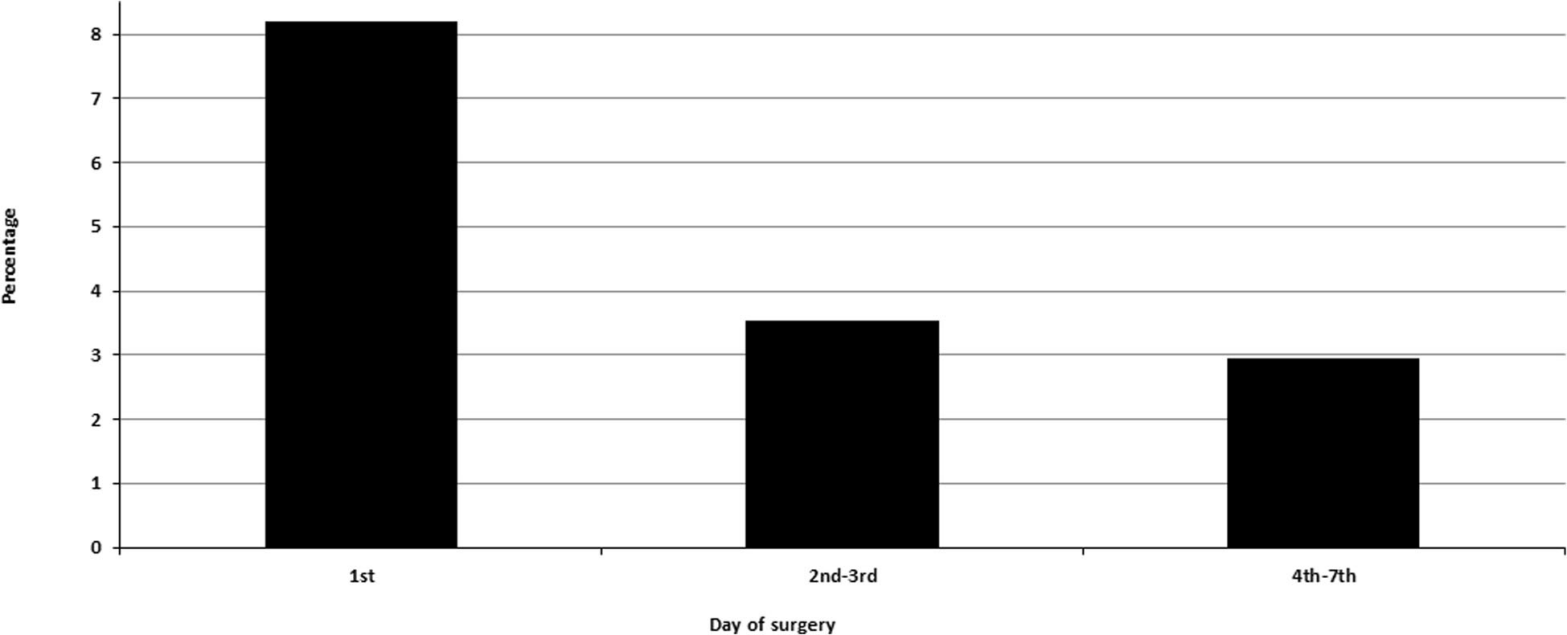
In-Hospital Mortality grouped by surgical timing

As shown on Fig. 2, crude hospital mortality rate after CABG within the first 24 h of STEMI (8.2%) was significantly greater than in group B (3.53%) and group C (2.94%, *P* < 0.0001 for both). This was confirmed in the multivariable logistic regression analysis with controlling for age, gender, race, the Elixhauser Comorbidity Index, and complications (group A vs B: OR = 1.85, 95%CI 1.52–2.25; group A vs C: OR = 2.21; 95%CI 1.82–2.68). No significant differences in hospital mortality between groups B and C were found both in Chi square test and multivariable logistic regression analysis.

To evaluate and compare hospital resource utilization, we calculated postoperative and total hospital LOS and total hospital cost. The Kolmogorov-Smirnov test for normality distribution found that all results were highly skewed to the right. For this reason, we presented them as median with the interquartile range (IQR) and compared with the non-parametric Wilcoxon rank sum test.

**Fig. 3:**
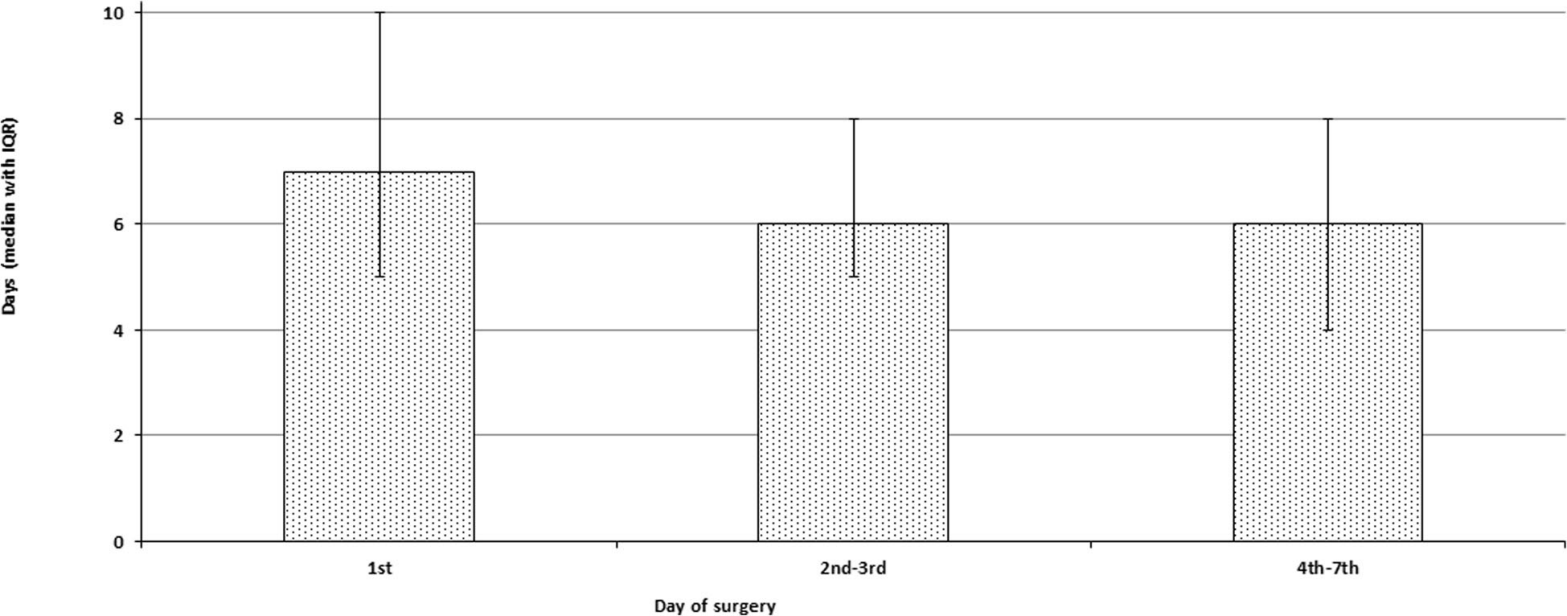
Post-Operative Hospital LOS grouped by surgical timing

**Fig. 4:**
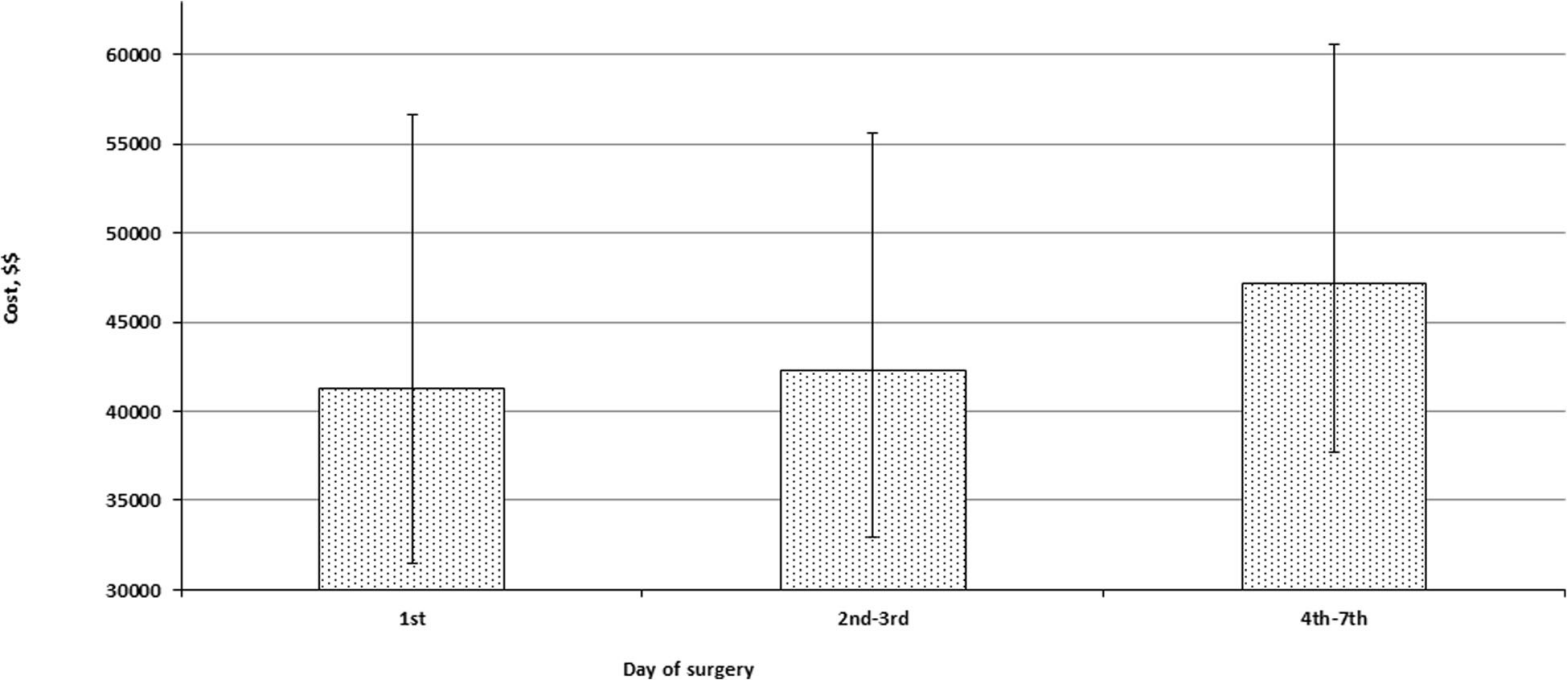
Total hospital cost grouped by surgical timing

As Fig. 3 demonstrates, the patients in group A had a significantly longer postoperative LOS (median 7 days with IQR 5–10 days) compared to those in group B (median 6 days, IQR 5–8 days) and group C (median 6 days, IQR 4–8 days; *P* < 0.0001 for both). Postoperative LOS in group B was also a little longer than in group C (*P* = 0.003). However, total hospital LOS in group A (median 7 days, IQR 5–10 days) was a little shorter than in group B (median 7 days, IQR 6–10 days; *P* < 0.0001) and considerably shorter than in group C (median 10 days, IQR 9–12 days; *P* < 0.0001). The difference between the latter two groups was also statistically significant (*P* < 0.0001). At the same time, as shown on Fig. 4, total hospital cost in group A (median $41,246; IQR $31,506-56,589) did not differ significantly from that in group B (median $42,322; IQR $32,969-55,620; *P* = 0.24) and both of them were significantly smaller than in group C (median $47.166; IQR $37,682–60.604; *P* < 0.0001 for both).

## Discussion

Acute STEMI usually results from coronary artery atherosclerotic plaque disruption with superimposed thrombus formation [5]. Primary percutaneous coronary intervention (PCI) is the preferred treatment for STEMI [2]. Approximately 20–30% of patients are not eligible for PCI and require surgical intervention [6] in the form of CABG. The data from our study shows that patients who develop STEMI and undergo CABG within the first 24 h have poor outcomes. They have significantly more complications and their mortality are more than twice for those patients who have their CABG delayed. These findings are important because cardiothoracic surgeons are frequently pressured to operate on these patients immediately. The data supports delaying surgical revascularization but not all surgical intervention. Although CABG is not recommended initially after STEMI there is a role for surgical intervention. The patients with STEMI can often develop acute cardiogenic shock and these patients can benefit from a left ventricular assist device (LVAD) such an Impella 5.0. The LVAD will function to decompress the left ventricle and allow the ventricle to recover.

In the United States, approximately 800,000 people annually are affected by AMI and in spite of a better awareness of presenting symptoms, about 225,000 die before reaching to the hospital [7]. In recent decades, improvements in treatment strategies have led to significant increase in survival rate for patients hospitalized with AMI. In the setting of AMI, the clinical spectrum may alter from STEMI to NSTEMI (subendocardial), or cardiogenic shock [8]. Surgery plays an important role in treatment of all these clinical scenarios because of advances in myocardial preservation and mechanical support. CABG for complete revascularization frequently may be put into practice as a therapeutic option in patients with NSTEMI. In contrast, the primary treatment for STEMI is PCI except for those patients who are not technically able to have it. The goal remains for the majority of patients with complex multivessel disease presenting with STEMI, to perform immediate percutaneous coronary intervention of the culprit vessel for revascularization. For the patients who require surgical revascularization for STEMI, those patients should have a delay in CABG to minimize morbidity and mortality.

The current American College of Cardiology/American Heart Association (**ACC/AHA**) and European revascularization guidelines for STEMI recommend culprit vessel revascularization during primary PCI as the primary intervention strategy in patients with multivessel disease [9, 10]. Despite these recommendations cardiothoracic surgeons are asked on many occasions to perform CABG on STEMI patients because of technical issues in the catherization laboratory. The findings from our study have been seen previously in a few studies but the debate on the timing of surgical revascularization is still present. Further support for our results has been stated beforehand and documented in the 2004 ACC/AHA guidelines. In the guidelines the management of patients with STEMI gives the following class IIa recommendation: “In patients who have had a STEMI, CABG mortality is elevated for the first 3 to 7 days after infarction, and the benefit of revascularization must be balanced against the increased risk. Patients who have been stabilized after STEMI and who have incurred a significant fall in LV function should have their surgery delayed to allow myocardial recovery to occur” [11]. The data from our study supports these statements and further helps to identify the optimal timing when patients should undergo CABG. Frequently, cardiothoracic surgeons do not have the luxury of waiting for 7 days to intervene and as a result reporting the impact of surgery earlier is important to know.

In this study, patients with STEMI who underwent CABG during the first 24 h after the event were found to have higher cardiac, respiratory and renal complications. They were also more likely to have increased in-hospital mortality and a longer length of hospital stay.

## Conclusions

The results of our study suggest that despite the urgency and severity of STEMI, a delay to surgical revascularization of at least 24 h may be beneficial to the short-term and long-term outcomes in these patients. If the STEMI patient is not hemodynamically stable then surgical intervention in the form of a LVAD would be appropriate.

## Data Availability

The data is available upon request.

## Abbreviations

STEMI: ST-Elevated Myocardial Infarction
AMI: Acute Myocardial Infarction
PCI: Percutaneous Coronary Infarction
MVD: Multivessel Coronary artery disease
CABG: Coronary Artery Bypass Grafting
AHRQ: Agency for Healthcare Research and Quality
HCUP: Healthcare Cost and Utilization Project
NIS: Nationwide/National Inpatient Sample
ACC/AHA: American College of Cardiology/American Heart Association
PRDAY1: Procedure day 1
